# Real-world drug survival and treatment outcome of ixekizumab in patients with spondyloarthropathies

**DOI:** 10.3389/fimmu.2026.1924119

**Published:** 2026-09-07

**Authors:** Lamia AL-Awuad, Munifah AL-Shammari, Nada Al-Dhayyan, Abdulrahman Alturaiki

**Affiliations:** 1 Pharmaceutical Care Services, Prince Mohammed bin Abdulaziz Hospital, Riyadh, Saudi Arabia; 2 King Abdulaziz Medical City, Pharmaceutical Care Services, Ministry of National Guard Health Affairs, Riyadh, Saudi Arabia; 3 College of Pharmacy, King Saud Bin Abdulaziz University for Health Sciences, Riyadh, Saudi Arabia; 4 King Abdullah International Medical Research Center, Riyadh, Saudi Arabia

**Keywords:** drug retention, interleukin-17, ixekizumab, psoriatic arthritis, real-world evidence, spondyloarthritis

## Abstract

**Background:**

Chronic inflammatory rheumatic diseases, such as ankylosing spondylitis, psoriatic arthritis, reactive arthritis, and enteropathic arthritis, are collectively referred to as spondyloarthropathies (SpA). However, Real-world evidence on the effectiveness and safety of ixekizumab in spondyloarthropathies remains limited, particularly in diverse populations outside clinical trial settings.

**Aim:**

This study aimed to evaluate patient demographics, treatment outcomes, drug survival, and safety profile of ixekizumab in patients with SpA in a real-world clinical setting.

**Methods:**

We conducted a retrospective cohort study at a tertiary care hospital in Riyadh, Saudi Arabia, from January 2018 to December 2024. Patients aged ≥14 years with SpA who received ixekizumab were included. The primary outcomes included drug survival, treatment discontinuation rates, and reasons for discontinuation. Kaplan-Meier survival analysis and Cox proportional hazards regression were performed to identify the predictors of treatment discontinuation.

**Results:**

Among 93 patients (mean age 44.2 ± 13.2 years, 60.2% female), psoriatic arthritis spectrum disorders predominated (71.0%), followed by ankylosing spondylitis (23.7%). Most patients (87.1%) had prior biologic exposure, with ixekizumab used predominantly as a second-line therapy (59.1%). Drug survival rates were 69.9% at 12 months and 43.0% at 24 months, with a median survival of 23.0 months (95% CI: 19.5-26.5). The overall discontinuation rate was 34.4%, primarily due to lack of efficacy (59.4%) and adverse events (18.8%). Treatment-emergent adverse events occurred in 7.5% of patients, with no serious adverse events reported. Disease type significantly influenced drug survival (p = 0.018), with psoriatic arthritis showing superior retention compared with ankylosing spondylitis (77.8% vs. 60.0% at 12 months). Multivariate analysis identified third-line or later therapy (HR 2.58, 95% CI 1.04-6.40, p = 0.041) and the presence of treatment-emergent adverse events (HR 9.86, 95% CI 3.42-28.41, p < 0.001) as independent predictors of discontinuation.

**Conclusion:**

Ixekizumab demonstrated acceptable drug survival and safety profiles in real-world patients with spondyloarthritis, with differential effectiveness across disease subtypes. Earlier treatment initiation and proactive management of adverse events may optimize treatment outcomes, supporting personalized therapeutic approaches for spondyloarthropathy management.

## Introduction

Spondyloarthropathies (SpA), which encompass disorders such as ankylosing spondylitis, psoriatic arthritis, reactive arthritis, and enteropathic arthritis, are chronic, long-term inflammatory rheumatic diseases (1). Recent epidemiological studies have shown that the prevalence of SpA varies significantly across the world’s regions, with some Southeast Asian populations reporting as low as 0.20% and Northern Arctic communities reporting 1.61% (2). This is strongly correlated with the patterns of HLA-B27 prevalence in the region. There is still a considerable burden of disease, with diagnostic delays averaging more than seven years in predicting the cases. The estimated market of USD 7.3 billion by 2033 shows the amount spent on healthcare, depicting the intertwined nature and high-burden cost that comes with managing chronic SpA, requiring extensive healthcare resources (3, 4).

The treatment landscape for SpA has undergone significant changes following the 2022 ASAS-EULAR recommendations, which emphasized more personalized medicine strategies (5, 6). Although biological DMARDs have revolutionized outcomes, NSAIDs continue to be the first-line therapy (6). Current options include Tumor necrosis factor (TNF)-inhibitors, Interleukin-17 (IL-17), IL-23, and JAK inhibitors. However, a recent meta-analysis demonstrated that TNF inhibitors remain the most efficacious in achieving ASAS responses, whereas IL-17 inhibitors show notable efficacy for BASDAI50 responses (7). Despite this progress, a significant non-responder rate of 20-40% persists, along with a loss of response over time, disparities in biological access worldwide, and exorbitant costs of originator biologics, ranging from £10, 000 to £80, 000 per year (8).

One of the most impactful advances in SpA therapeutics is ixekizumab, a high-affinity humanized IgG4 monoclonal antibody that targets IL-17A (9, 10). Ixekizumab’s picomolar affinity for IL-17A facilitates its role as an antagonist by preventing inflammatory processes essential to SpA from spiraling out of control (11). The FDA authorized ixekizumab for the treatment of psoriatic arthritis in 2017, ankylosing spondylitis in 2019, and, crucially, as the first IL-17A antagonist for non-radiographic axial spondyloarthritis in 2020 (11). Evidence now supports dosing regimens starting with a loading dose of 160 mg, followed by 80 mg every four weeks, with sustained efficacy demonstrated over 156 weeks (12).

Clinical efficacy results from phase 3 pivotal trials have demonstrated strong results throughout the SpA spectrum. In the COAST Program for axial SpA, ASAS40 response rates were 35-52% compared to 18-19% in placebo participants at week 16, with responses maintained for three years (13–15). In psoriatic arthritis, the SPIRIT trials reported ACR20 responses of 48-62%, compared to 20-30% in placebo responders, with remarkable enthesitis resolution in 39% and dactylitis resolution in 78% (14, 16, 17). Long-term follow-up also revealed no new concerns, apart from mild injection-site reactions and infections (18–20).

Over time, treatment outcomes have become increasingly linked to patient demographics. In HLA-B27-positive patients, the retention and response rates of TNF inhibitors are significantly higher (21, 22). Age, sex, BMI, and smoking status are among the factors that have a marked impact on therapeutic efficacy. Recently emerging troubling evidence shows clinical trial-wide demographic disparities, where White participants composed 82%, rendering these findings unhelpful for generalizing them to real-life diverse patient populations (23).

Nevertheless, there is still a lack of evidence based on real-world studies, such as long-term outcome data concerning elderly patients, ethnic minorities, or those with multiple comorbidities, who are systematically excluded from clinical trials. The focus on conducting clinical research on Western populations limits our understanding of how individuals with diverse genetic backgrounds and healthcare systems respond to treatment, thereby deepening this knowledge gap. Therefore, our study aims to fill these gaps by evaluating demographic data alongside treatment data of ixekizumab in real-world populations to inform policy on enhancing personalized treatment approaches that advance equity within the SpA disorder management frameworks.

## Methods

### Study design and setting

This retrospective cohort study was conducted at the King Abdulaziz Medical City (KAMC) and King Abdullah Specialized Children’s Hospital in Riyadh, Saudi Arabia. Both are tertiary care facilities within the National Guard Health Affairs (NGHA) system. Our study aimed to assess the real-world effectiveness, safety, and drug survival of ixekizumab in patients with spondyloarthropathies between January 2018 and December 2024.

### Study population and eligibility criteria

Eligible participants were those who were 14 years of age at treatment initiation with an appropriate medically documented history of spondyloarthropathy fulfilling the ASAS criteria for axial spondyloarthritis, the Classification Criteria for Psoriatic Arthritis (CASPAR) for psoriatic arthritis, or the modified New York criteria for ankylosing spondylitis. Furthermore, they needed to have received a treatment course of ixekizumab during the study timeline with at least 3 months of follow-up time post-treatment start, along with electronic health records capturing relevant baseline details and outcomes documenting treatment results.

Patients were excluded from the study if they had incomplete medical records due to loss to follow-up within three months of treatment initiation, concurrent participation in other clinical trials during the study timeframe, or changes in diagnosis during follow-up that no longer classified them as having spondyloarthropathy. A total of 33 patients were excluded based on these criteria, including 3 patients whose diagnosis was revised during follow-up. These criteria were established to safeguard the integrity of data collection and minimize any potential bias that could impact the evaluation of real-world effectiveness presented in this study.

### Sample size calculation

Considering prior reports indicating that ixekizumab discontinuation rates range between 30-40% after one year, we estimated that a sample size of 93 patients would achieve 80% power to detect clinically significant differences in drug survival rates with a two-sided alpha of 0.05, assuming a drug survival rate of 65% at one year. This was calculated using standard formulas for survival analysis sample sizes, considering the event rate and duration of the follow-up period expected in real-world biological therapy studies.

### Data collection methods and sources

The electronic health record (EHR) system served as the primary source of data for this study, consolidating complete medical record information from all NGHA facilities. A standardized data collection form was created and piloted on ten patients to evaluate its efficacy in terms of completeness and accuracy, before broader implementation.

### Study variables and measurements

Demographic data, including age at treatment initiation, sex, weight, height, and BMI classification, were recorded. Clinically relevant information included a primary spondyloarthropathy diagnosis, along with its associated disease subtype, which is often linked to other medical conditions such as diabetes mellitus, hypertension, dyslipidemia, fibromyalgia, ischemic heart disease, and chronic kidney disease. Information about HLA-B27 status was documented if it had been previously noted in the patients’ charts, although it was not a routine test performed for almost all patients.

The baseline inflammatory markers for the laboratory tests included C-reactive protein (CRP, with normal values of less than 3 mg/L) and erythrocyte sedimentation rate (ESR, with normal values of less than 20 mm/h for females and less than 15 mm/h for males). Renal function was assessed using the estimated glomerular filtration rate (eGFR), determined by the Chronic Kidney Disease Epidemiology Collaboration (CKD-EPI), which is the most accurate estimator of kidney function in different populations.

Treatment history and characteristics involved recording the line of biologic therapy as first-line biologic-naïve therapy or second-line and later therapies. Prior exposure to biologics was systematically classified according to their mechanism of action, including TNF, IL-17, IL-23, and JAK inhibitors, as well as other biologic agents. Other medications included conventional disease-modifying antirheumatic drugs (DMARDs), nonsteroidal anti-inflammatory drugs (NSAIDs), and oral corticosteroids, which could modify treatment outcomes.

### Outcome definitions and assessments

The primary outcomes measured included treatment duration and drug survival, which was defined as the interval from initiation of ixekizumab to discontinuation of therapy for any reason. Patients who continued treatment were censored at their last follow-up visit. The documented reasons for discontinuation were systematically classified as lack of efficacy, adverse events, non-compliance by the patient, pregnancy, or other medical reasons, with available evidence noted in the charts.

Treatment-emergent adverse events (TEAEs) were defined as any detrimental health issue that arose or worsened following the use of ixekizumab, regardless of whether it was related to the study drug. TEAEs were categorized using the Medical Dictionary for Regulatory Activities terminology framework to ensure uniformity in reporting. Serious adverse events were restricted to those comprising life-threatening conditions, hospitalization, persistent or significant disability with long-lasting functional impact, or death. The continuity status of drug survival and persistence patterns was evaluated during the last follow-up visit.

### Statistical analysis plan

All statistical analyses were conducted using SAS Statistics version 9.4. Continuous variables that followed a normal distribution were summarized using the mean and standard deviation, whereas non-normally distributed variables were summarized using the median and interquartile range (IQR). Categorical variables were summarized as frequencies and percentages, with the relevant denominators clearly stated. Missing data patterns were systematically evaluated, including descriptions, and analyses were conducted on complete datasets with appropriate denominators reported for all relevant variables.

Between-group comparisons were conducted using appropriate statistical tests, aligned with the underlying data distribution. For continuous variables, normality was assessed using the Shapiro-Wilk test to inform the choice of appropriate statistical methods for comparative analyses. However, for continuous variables that are normally distributed, independent samples t-tests are applicable; for continuous variables that are not normally distributed, the Mann-Whitney U test is more suitable. Categorical variables were subjected to either chi-square tests or Fisher’s exact tests. Fisher’s exact test was used when the expected counts for cells were below five to maintain the integrity of the hypothesis testing framework.

Drug survival was analyzed using the Kaplan-Meier method of survival analysis. Overall, drug survival curves were generated for the entire cohort to evaluate the treatment persistence trends within the population. Stratified analyses by disease, line of therapy, sex, and concomitant medications were conducted to identify possible factors affecting drug survival. Log-rank tests were applied to compare the survival curves of the different subgroups, and median survival times were calculated with 95% confidence intervals to provide clinically relevant estimates.

Treatment discontinuation risk was assessed in a univariate analysis using logistic regression, which identified potential risk factors. The results were presented as odds ratios (OR) with 95% confidence intervals. In consideration of these models, only variables with p-values less than 0.10 in univariate testing were included in the multivariate modeling aimed at controlling for confounding factors.

Multivariate analysis employed Cox proportional hazards regression modeling to identify independent risk factors for treatment discontinuation, taking into account relevant covariates. Two sequential models were constructed to capture the different dimensions of risk prediction. Model 1 included only clinical variables: age, sex, disease type, and line of therapy. Variables that did not demonstrate an independent association with discontinuation in Model 1 were not retained in the fully adjusted model to avoid overfitting in this modest-sized cohort. Model 2 was built upon the first model by integrating treatment-emergent adverse events to assess their independent contribution to the risk of discontinuation.

For all analyses performed in this study, a two-sided p-value less than 0.05 was deemed statistically significant. Owing to the exploratory nature of the subgroup analyses and the observational design of the study, no adjustments for multiple comparisons were made. For complete clinical interpretation, effect sizes and confidence intervals were calculated and presented alongside the reported p-values.

### Ethics approval and considerations

Adhering to the Declaration of Helsinki guidelines and international ethical standards for medical research, this investigation followed the strict protocols of Good Clinical Practice. Before commencing the study, a moral application was submitted and approved by the Institutional Review Board of the King Abdullah International Medical Research Center (approval number 00000109925). Considering that this study was retrospective (i.e., using preexisting data), the ethics board determined that obtaining informed consent was not necessary, as only coded data from clinical routines were used in the study. As part of this study, all patient records underwent systematic de-identification before any analysis was conducted, ensuring that no identifiable information could be linked to a separate database. Access to the encrypted electronic files, which were stored on Secure NGHA Servers, was strictly limited to trained researchers with the appropriate Clearance Level and data protection training, who had been given explicit approval for access. Protective measures for data storage applied at both the physical and electronic levels adhered to institutional policies aimed at safeguarding sensitive information.

## Results

### Demographic and clinical characteristics at baseline

Our study included 93 patients with spondyloarthropathy, with a mean age of 44.2 ± 13.2 years (range, 16–71 years) and female predominance (60.2% vs. 39.8% male). While this distribution diverges from the classical epidemiology of ankylosing spondylitis, it aligns with patterns observed in psoriatic arthritis, which is more representative of our cohort. Regarding anthropometric data, the metabolic profiles suggested a noteworthy mean BMI of 30.0 ± 6.7 kg/m², classifying most patients as obese. Other comorbid disorders, such as diabetes mellitus (23.7%), hypertension (22.6%), and dyslipidemia (21.5%), far surpassed the rates among the general population, indicating the inflammatory burden typical of spondyloarthropathies. Additional comorbidities included fibromyalgia (6.5%), ischemic heart disease (3.2%), and chronic kidney disease (1.1%). Laboratory measures reflected active inflammation with substantial disease activity at treatment initiation, evidenced by a markedly elevated mean CRP of 18.5 ± 58.6 mg/L (normal <3 mg/L) and ESR of 38.3 ± 26.4 mm/hr above normal (<20 mm/hr for women, <15 for men). Additionally, their renal function was preserved (eGFR 104.6 ± 20.6 mL/min/1.73 m²) (**Table 1**).

**Table 1:** Baseline demographics and clinical characteristics of patients treated with ixekizumab (N = 93).

Characteristic	Value
Age, years	
Mean ± SD	44.2 ± 13.2
Range	16-71
Sex, n (%)	
Male	37 (39.8)
Female	56 (60.2)
Anthropometric measures	
Weight, kg, mean ± SD	80.0 ± 21.5
Height, cm, mean ± SD	160.0 ± 16.4
Body mass index, kg/m², mean (SD)^a^	30.0 (6.7)
Comorbidities, n (%)	
Diabetes mellitus	22 (23.7)
Hypertension	21 (22.6)
Dyslipidemia	20 (21.5)
Fibromyalgia	6 (6.5)
Ischemic heart disease	3 (3.2)
Chronic kidney disease	1 (1.1)
Other comorbidities	20 (21.5)
Laboratory values	
C-reactive protein, mg/L, mean ± SD (n=55)	18.5 ± 58.6
eGFR, mL/min/1.73m², mean ± SD (n=86)	104.6 ± 20.6
Erythrocyte sedimentation rate, mm/hr (n=69)	38.3 (26.4)

SD, standard deviation; eGFR, estimated glomerular filtration rate, ^a^Based on available height and weight data,

### Disease characteristics and treatment indications

The distribution of diseases reflected the epidemiology of spondyloarthropathy, where disorders within the spectrum of psoriatic arthritis predominated (71.0%, n=66) and included isolated psoriatic arthritis (38.7%, n=36), psoriasis with concurrent psoriatic arthritis (31.2%, n=29), and rare axial involvement (1.1%, n=1). Ankylosing spondylitis accounted for 23.7% (n = 22) of the cases, primarily as an isolated disease (21.5%, n = 20) with minimal overlap with psoriatic features (2.2%, n = 2). Other spondyloarthropathies contributed to 3.2% (n=3), including juvenile idiopathic arthritis (2.2%, n=2) and seronegative spondyloarthropathy of unknown subtype (2.2%, n=2). Additionally, hidradenitis suppurativa and psoriasis were reported separately (1.1%, n = 1 each), as shown in **Table 2**.

**Table 2:** Disease characteristics and treatment indications.

Primary indication	n (%)
**Psoriatic arthritis spectrum**	66 (71.0)
- Psoriatic arthritis only	36 (38.7)
- Psoriasis with psoriatic arthritis	29 (31.2)
- PsA with axial spondyloarthritis	1 (1.1)
**Ankylosing spondylitis spectrum**	22 (23.7)
- Ankylosing spondylitis only	20 (21.5)
- AS with psoriasis/PsA	2 (2.2)
**Other spondyloarthropathies**	3 (3.2)
- Juvenile idiopathic arthritis	2 (2.2)
- Unspecified/seronegative SpA	2 (2.2)
**Other indications**	2 (2.2)
- Hidradenitis suppurativa	1 (1.1)
- Psoriasis only	1 (1.1)

Bold text indicates main categories; indented entries represent subcategories where applicable.

### Treatment characteristics

Based on the treatment pattern analysis, ixekizumab was primarily used as a last-resort intervention in patients who had already undergone rigorous treatment, with only 12.9% (n = 12) being biologic-naïve and 87.1% (n = 81) having prior exposure to biologic therapy. Second-line therapy was the most common (59.1%, n=55), followed by third-line (25.8%, n=24) and fourth-line use (2.2%, n=2), indicating sequential biologic failure and positioning following Tumor necrosis factor (TNF)-inhibitor inadequacy. Among these previously treated patients, the lone prior TNF inhibitor-exposed patient group was the most dominant (46.9%, n=38), comprising patients treated with adalimumab, etanercept, and infliximab. In contrast, the IL-17 inhibitor group (19.8%, n=16) suggested intra-class switching to IL-17-dependent pathways. Multiple drug classes were involved in 28.4% of cases (n=23), signifying treatment-resistant disease, where other biologics included but were not limited to 4.9% (n=4) IL-23 inhibitors, JAK inhibitors, PDE4 inhibitors, or even abatacept. Concomitant traditional therapy was commonplace: DMARDs/NSAIDs were prescribed to over half of the participants (60.2%, n=56), while prescription for oral corticosteroids remained low at 12.9% (n=12). However, the critical gap in HLA-B27 testing (88.2% not tested/unknown, 9.7% negative, 2.2% positive) represents a significant limitation in genetic characterization that may impact treatment response prediction and personalized therapy approaches, particularly given HLA-B27’s established role in axial spondyloarthritis pathogenesis and therapeutic response, as shown in **Table 3**.

**Table 3:** Treatment characteristics.

Characteristic	n (%)
Line of therapy	
First line (biologic-naïve)	12 (12.9)
Second line	55 (59.1)
Third line	24 (25.8)
Fourth line	2 (2.2)
Concomitant medications	
Conventional DMARD/NSAID	56 (60.2)
Oral corticosteroids	12 (12.9)
Previous biologic exposure (n=81)	
TNF inhibitors only^c^	38 (46.9)
IL-17 inhibitors only^d^	16 (19.8)
Multiple biologic classes	23 (28.4)
Other biologics^e^	4 (4.9)
HLA-B27 Status	
Not tested/Unknown	82 (88.2)
Negative	9 (9.7)
Positive	2 (2.2)

DMARD, disease-modifying antirheumatic drug; NSAID, non-steroidal anti-inflammatory drug; ^a^Includes adalimumab, etanercept, and infliximab ^b^Primarily secukinumab; ^c^Includes IL-23 inhibitors (risankizumab), JAK inhibitors (tofacitinib), PDE4 inhibitors (apremilast), and abatacept.

### Drug survival and discontinuation outcomes

Drug survival analysis revealed modest retention rates, which are typical of real-life biological practices in treating our populations (**Table 4**). At the 12-month mark, 69.9% (n = 65/93) of participants maintained treatment, which decreased to 43.0% (n = 40/93) at the 24-month mark, with a median survival of 23.0 months (IQR, 12.0-35.5). Overall, the discontinuation rate was 34.4% (n = 32) among patients. Specific patterns related to the disease emerged: patients with psoriatic arthritis had the lowest discontinuation rate (31.8%, n = 21/66), followed by those with ankylosing spondylitis (40.9%, n = 9/22) and other spondyloarthropathies (40.0%, n = 2/5). The average treatment duration of 24.7 ± 15.2 months suggests a bimodal distribution, with early responders experiencing early termination due to ineffectiveness or intolerance, in contrast to sustained long-term use among true responders and prolonged use among those who truly benefit from the treatment.

**Table 4:** Drug survival and discontinuation outcomes.

Outcome	n (%)
Treatment status at last follow-up	
Continued Ixekizumab	61 (65.6)
Discontinued Ixekizumab	32 (34.4)
Discontinuation by indication group	
Psoriatic arthritis patients (n=66)	21/66 (31.8)
Ankylosing spondylitis patients (n=22)	9/22 (40.9)
Other indications (n=5)	2/5 (40.0)
Drug survival rates	
At 12 months, n/N (%)	65/93 (69.9)
At 24 months, n/N (%)	40/93 (43.0)
Treatment duration	
Mean (SD), months	24.7 (15.2)
Median (IQR), months	23.0 (12.0-35.5)

### Reasons for discontinuation

For the 32 patients (34.4%) who discontinued ixekizumab treatment, lack of effectiveness was the predominant reason (59.4%, n = 19), followed by adverse events (18.8%, n = 6). This illustrates how therapeutic effectiveness often struggles in real-world settings, where safety concerns prevail. Other reasons for discontinuation included non-compliance with therapy (3.1%, n=1), pregnancy (3.1%, n=1), and other medical reasons, such as an ear infection necessitating treatment discontinuation (3.1%, n=1). Notably, 12.5% (n = 4) did not explain, indicating sparse documentation in clinical practices (**Table 5**).

**Table 5:** Reasons for discontinuation.

Outcome	Value
Reasons for discontinuation (n=32)	
Lack of efficacy	19 (59.4)
Adverse events	6 (18.8)
Patient non-compliance	1 (3.1)
Pregnancy	1 (3.1)
Other reasons^c^	1 (3.1)
Not specified	4 (12.5)

^c^Includes ear infection requiring treatment discontinuation.

### Treatment-emergent adverse events

The safety profile reflected good tolerability, as treatment-emergent adverse events occurred in 7.5% (n = 7) of the patients. Notably, there were no serious adverse events (**Table 6**). The most common adverse reaction to the treatment was injection site reactions (4.3%, n=4), which aligns with the subcutaneous route of administration and is similar to those of other IL-17 antagonists. Infectious complications were rare, including upper respiratory tract infection (1.1%, n=1) and urinary tract infection (1.1%, n=1), resulting in a combined infection rate of 2.2%. Dermatological reactions, such as skin rash and pruritus, occurred in of 1.1% (n = 1).

**Table 6:** Treatment-emergent adverse events (TEAEs).

Adverse event	n (%)
Any TEAE	7 (7.5)
Specific TEAEs	
Injection site reaction	4 (4.3)
Upper respiratory tract infection	1 (1.1)
Urinary tract infection	1 (1.1)
Skin rash and pruritus	1 (1.1)
Serious Adverse Events (SAEs)	0 (0.0)

### Subgroup analysis of drug survival by line of therapy

Drug survival was inversely correlated with the treatment line, illustrating the principle of diminishing returns associated with sequential biologic therapy. Biologic-naïve first-line patients had significantly better retention rates (83.3% at 12 months, 58.3% at 24 months, n = 12) than second-line (69.1% and 41.8%, n = 55) and third-line (66.7% and 37.5%, n = 24) patients. Fourth-line patients had a lower survival than all other groups at both time points (50.0%, n = 2), but were hindered by a small sample size (**Table 7**).

**Table 7:** Subgroup analysis of drug survival by line of therapy.

Line of therapy	N	12-month survival, n (%)	24-month survival, n (%)
First line (biologic-naïve)	12	10 (83.3)	7 (58.3)
Second line	55	38 (69.1)	23 (41.8)
Third line	24	16 (66.7)	9 (37.5)
Fourth line	2	1 (50.0)	1 (50.0)

### Comparison of baseline characteristics between discontinued vs. continued patients

Association analysis revealed no significant differences in baseline characteristics across several domains between patients who discontinued (n = 32) and those who continued (n = 61) treatment with ixekizumab (**Table 8**). Their demographic features were comparable, including the mean age (43.5 ± 12.2 vs. 44.5 ± 13.8 years, p = 0.734), proportion of females (62.5% vs. 59.0%, p = 0.744), and BMI (29.8 ± 6.5 vs. 30.1 ± 6.8 kg/m², p = 0.842). The absence of significant differences extended to the comorbidity profile, including diabetes mellitus (28.1% vs. 21.3%, P = 0.458), hypertension (25% vs. 21.3%, P = 0.684), dyslipidemia (25% vs. 19.7%, P = 0.547), and fibromyalgia (9.4% vs. 4.9%, P = 0.398). A similar distribution of primary indications for disease characteristics was observed (p = 0.612), along with comparable treatment histories regarding the line of therapy (p = 0.520), concomitant medication use with DMARDs/NSAIDs (p = 0.522), and oral steroids (p = 0.511). The lack of significance was observed for the baseline inflammatory markers CRP (8.5 vs. 5 mg/L, p = 0.089) and ESR (38.0 vs. 32.0 mm/hr, p = 0.214).

**Table 8:** Comparison of baseline characteristics between patients who discontinued vs. continued ixekizumab treatment.

Characteristic	Discontinued (n=32)	Continuing (n=61)	p-value
Demographics			
Age, years, mean ± SD	43.5 ± 12.2	44.5 ± 13.8	0.734^a^
Female sex, n (%)	20 (62.5)	36 (59.0)	0.744^b^
Body mass index, kg/m², mean ± SD	29.8 ± 6.5	30.1 ± 6.8	0.842^a^
Comorbidities, n (%)			
Diabetes mellitus	9 (28.1)	13 (21.3)	0.458^b^
Hypertension	8 (25.0)	13 (21.3)	0.684^b^
Dyslipidemia	8 (25.0)	12 (19.7)	0.547^b^
Fibromyalgia	3 (9.4)	3 (4.9)	0.398^c^
Disease characteristics			
Primary indication, n (%)			0.612^b^
Psoriatic arthritis	23 (71.9)	42 (68.9)	
Ankylosing spondylitis	9 (28.1)	13 (21.3)	
Other spondyloarthropathies	0 (0.0)	6 (9.8)	
Baseline CRP, mg/L, median (IQR)^d^	8.5 (3.0-19.0)	5.0 (2.0-12.0)	0.089^e^
Baseline ESR, mm/hr, median (IQR)^d^	38.0 (22.0-55.0)	32.0 (18.0-48.0)	0.214^e^
Treatment History			
Line of therapy, n (%)			0.520^b^
First line (biologic-naïve)	3 (9.4)	9 (14.8)	
Second line or later	29 (90.6)	52 (85.2)	
Concomitant DMARDs/NSAIDs, n (%)	19 (59.4)	32 (52.5)	0.522^b^
Concomitant oral steroids, n (%)	7 (21.9)	10 (16.4)	0.511^b^

^a^Independent t-test; ^b^Chi-square test; ^c^Fisher’s exact test; ^d^Based on available data; ^e^Mann-Whitney U test. Bold text indicates main categories; indented entries represent subcategories where applicable.

### Analysis of drug survival rates by patient subgroups

The subgroup analysis revealed marked heterogeneity in treatment effectiveness, with the type of disease emerging as a significant factor influencing drug survival (p = 0.018) (**Table 9**). Patients with psoriatic arthritis demonstrated better retention (77.8% at 12 months, 47.6% at 24 months, median survival 24.5 months, n=63) compared to those with ankylosing spondylitis (60.0%, 40.0%, 18.0 months, n=20) and other spodyloarthritis (40%, 20%, 10.5months, n=10). These differing responses may be attributed to the role of IL-17A in peripheral joint and skin inflammation in psoriatic arthritis versus the axial inflammation that is dominant in ankylosing spondylitis pathology. Line of therapy also had a significant effect on results (p=0.042), first line therapy provided best outcomes (83.3%, 58.3%, 30.0 months, n=12) compared to second line (69.1%, 41.8%, 22 months, n=55) and third or later line therapy (65.4%, 38.5%, 19.5 months, n=26). Importantly, sustained DMARD/NSAID co-therapy did not result in better survival rates (72.5% vs. 66.7% at year one, p = 0.465). Statistical significance was not observed for gender differences (p = 0.287); however, a numerically better response was observed among males (75.7% vs. 66.1% at 12 months).

**Table 9:** Drug survival analysis by patient subgroups.

Subgroup	N	12-Month survival n (%)	24-Month survival n (%)	Median survival (months)	p-value*
Overall cohort	93	65 (69.9)	40 (43.0)	23.0	—
By disease type					0.018
Psoriatic arthritis	63	49 (77.8)	30 (47.6)	24.5	
Ankylosing spondylitis	20	12 (60.0)	8 (40.0)	18.0	
Other spondyloarthropathies	10	4 (40.0)	2 (20.0)	10.5	
By line of therapy					0.042
First line (biologic-naïve)	12	10 (83.3)	7 (58.3)	30.0	
Second line	55	38 (69.1)	23 (41.8)	22.0	
Third line or later	26	17 (65.4)	10 (38.5)	19.5	
By concomitant therapy					
With DMARDs/NSAIDs	51	37 (72.5)	24 (47.1)	24.0	0.465
Without DMARDs/NSAIDs	42	28 (66.7)	16 (38.1)	21.0	
By gender					
Female	56	37 (66.1)	22 (39.3)	22.0	0.287
Male	37	28 (75.7)	18 (48.6)	24.5	

*Log-rank test for survival curve comparison.

### Univariate analysis of factors associated with treatment discontinuation

The unprotected trends and specific risk factors associated with treatment discontinuation were highlighted in the analysis, as shown in **Table 10**. Therapy provided in the third position or later appeared to be a more prominent risk factor (OR 2.45, 95% CI 1.01-5.95, p = 0.048), which corroborates the evidence of waning effectiveness over sequential biologic attempts and supports early intervention strategies. The presence of treatment-emergent adverse events strongly predicted discontinuation (OR 11.54, 95% CI 1.32-100.9, p = 0.027), although the wide confidence interval suggested a relatively low event rate (7.5%). Age > 60 years showed a protective trend (OR 0.22, 95% CI 0.03-1.81, p = 0.149). A borderline association was observed between elevated CRP levels (greater than 10 mg/L) and discontinuation (OR 2.18, 95% CI 0.87-5.46, p = 0.096). The analyzed demographic variables, including female sex (OR 1.16, p = .744) and obesity defined by a body mass index (BMI) of 30 kg/m² or greater (OR 0.92; p = .848), yielded no significant results. The absence of comorbidity was also found not to be predictive, with values such as (OR 1.31, p = 0.542). Diabetes (OR 1.44, p = 0.459) did not exhibit confirmatory evidence within the theme. Neither disease-related consideration of ankylosing spondylitis versus psoriatic arthritis (odds ratio [OR] 1.27, p = 0.635) nor prior biologic therapy experience compared with naïve status (OR 1.67, p = 0.461) was statistically significant.

**Table 10:** Univariate analysis of factors associated with treatment discontinuation.

Risk factor	Odds ratio (95% CI)	p-value
Demographics		
Female sex	1.16 (0.48-2.78)	0.744
Age ≥ 60 years	0.22 (0.03-1.81)	0.149
BMI ≥ 30 kg/m²	0.92 (0.39-2.17)	0.848
Comorbidities		
Presence of any comorbidity	1.31 (0.55-3.11)	0.542
Diabetes mellitus	1.44 (0.54-3.86)	0.459
Hypertension	1.23 (0.45-3.36)	0.684
Disease-related factors		
Ankylosing spondylitis (vs. PsA)	1.27 (0.48-3.38)	0.635
Elevated baseline CRP (>10 mg/L)	2.18 (0.87-5.46)	0.096
Treatment-related factors		
Biologic-experienced (vs. naïve)	1.67 (0.43-6.53)	0.461
Third-line or later therapy	2.45 (1.01-5.95)	0.048
No concomitant DMARDs	1.32 (0.56-3.08)	0.522
Adverse events		
Presence of any TEAE	11.54 (1.32-100.9)	0.027

### Treatment-emergent adverse events and their relationship with drug discontinuation

The evaluation of treatment-emergent adverse events revealed a strong association between safety events and treatment discontinuation, which is notable given the overall acceptable safety profile, as shown in **Table 11**. Out of the 7 patients (7.5%) who experienced any treatment-emergent adverse event, 6 (85.7%) discontinued therapy. This discontinuation resulted in a relative risk of 2.84 (95% CI, 1.81-4.45; p = 0.003) compared with individuals who did not experience any complications. The observed high rate of discontinuation among patients experiencing adverse events underscores the need for comprehensive early safety evaluations, coupled with active management strategies, to address potential adverse events. Injection site reactions, the predominant adverse event in injection site reactions seen in 4 patients (4.3%), resulted in discontinuation in 3 out of 4 cases (75%). This resulted in a relative risk of 2.31 (95% CI, 1.19-4.48; p = 0.089). Although this association was close to statistical significance, the considerable rate of discontinuation suggests that even minor local reactions can dramatically influence treatment adherence, indicating either patient tolerance thresholds or inadequate expectations management, as well as the need for counseling regarding expectable reactions at the injection sites. Infectious complications, although infrequent (2.2%, n = 2), posed the greatest risk of therapy discontinuation, as both affected patients discontinued therapy (RR 3.12, 95% CI 2.31-4.21, p = 0.046). This observation is consistent with clinical practice guidelines, which recommend pausing therapy in the event of significant infections and emphasize the importance of prompt infection control and recognition of the infection. The sole case of a skin reaction (1.1%) also resulted in 100% discontinuation (RR 3.12, 95% CI 2.16-4.50, p = 0.344), which lacked significance due to the small sample size.

**Table 11:** Treatment-emergent adverse events and their association with discontinuation.

Adverse event	Total events n (%)	Led to discontinuation n (%)	RR (95% CI)^a^	p-value
Any TEAE	7 (7.5)	6 (85.7)	2.84 (1.81-4.45)	0.0003^f^
Specific TEAEs				
Injection site reaction	4 (4.3)	3 (75.0)	2.31 (1.19-4.48)	0.089
Infections	2 (2.2)	2 (100.0)	3.12 (2.31-4.21)	0.046
Skin reactions	1 (1.1)	1 (100.0)	3.12 (2.16-4.50)	0.344
**Serious AEs**	0 (0.0)	—	—	—

^a^Relative Risk of discontinuation compared to patients without the specific adverse event ^f^Fisher’s exact test.

Bold text indicates category headings for adverse events.

### Multivariate Cox regression analysis for predictors of treatment discontinuation

From the analysis conducted using multivariate Cox regression, several independent predictors of treatment discontinuation were identified after controlling for potential confounding variables (**Table 12**). When considering clinical parameters only in Model 1, we found that therapy given third-line or later was a strong predictor (HR 2.67, 95% CI 1.08-6.60, p = 0.033), confirming that outcome independence by patient factors captures characteristics beyond those. Age showed no significant association (HR 0.92 per decade of age), 95% CI 0.68-1.24, p = 0.574), which contradicts the protective trend of aging noted in the univariate analysis. This suggests that mediation through other factors, such as comorbid conditions or expectations regarding treatment, may explain the effects of age. Sex did not have a significant impact on treatment outcomes (HR 1.18, 95% CI 0.57-2.44, p = 0.656), supporting the absence of sex-based discrimination in treatment outcomes. Having ankylosing spondylitis rather than other diagnoses displayed a trend towards a higher risk of discontinuation, which was not statistically significant (HR 1.42, 95% CI 0.64-3.15, p = 0.389). This suggests that some prior differences may become less pronounced after accounting for other factors.

**Table 12:** Multivariate Cox regression analysis for predictors of treatment discontinuation.

Variable	Hazard ratio (95% CI)	p-value
Model 1: Clinical factors		
Age (per 10 years)	0.92 (0.68-1.24)	0.574
Female sex	1.18 (0.57-2.44)	0.656
Ankylosing spondylitis	1.42 (0.64-3.15)	0.389
Third-line or later therapy	2.67 (1.08-6.60)	0.033
Model 2: Including adverse events		
Age (per 10 years)	0.89 (0.66-1.21)	0.465
Female sex	1.21 (0.58-2.52)	0.612
Third-line or later therapy	2.58 (1.04-6.40)	0.041
Presence of TEAE	9.86 (3.42-28.41)	< 0.001

Within model two, with the inclusion of adverse event(s), treatment-emergent adverse event(s) stood out as the strongest predictor of discontinuation (HR 9.86, 95% CI 3.42-28.41, p<0.001) events, marking approximately a tenfold increased hazard over patients without such events. Notably, the inclusion of adverse events in the model only slightly attenuated the effect of third-line therapy (HR 2.58, 95% CI 1.04-6.40, p = 0.041), suggesting that treatment order and safety events have independent impacts on survival. The substantial hazard for adverse events underscores their critical effect on treatment adherence, suggesting that more attention should be devoted to these areas to enhance ixekizumab outcomes.

### Reasons for discontinuation stratified by key variables

Analysis of the reasons for discontinuation revealed some significant clinical findings, although statistical significance was hindered due to smaller sample sizes (**Table 13**). Of the thirty-two patients who discontinued ixekizumab, the predominant reason cited by the majority (59%, n = 19) was a lack of efficacy. Patients with psoriatic arthritis (n = 23) demonstrated a moderate efficacy-related discontinuation rate of 52.2% (n = 12), while those with ankylosing spondylitis (n = 9) had a significantly higher rate of 77.8% (n = 7). Although this difference did not reach statistical significance (p = 0.238), it could indicate differential involvement of the IL-17A pathway in disease pathophysiology, where axial inflammation may be less responsive to IL-17A inhibition than psoriatic peripheral joint and skin manifestations, which respond more robustly clinically. The higher failure rate reported in ankylosing spondylitis is consistent with ASAS response rates compared to ACR responses in psoriatic arthritis, suggesting different mechanisms in therapeutic aims. Discontinuations due to adverse events were noted in 21.7% (n = 5) of patients with psoriatic arthritis versus 11.1% (n = 1) of patients with ankylosing spondylitis (p = 0.652), indicating comparable tolerability across these disease types and suggesting different adverse event profiles for these medications. All other reasons for discontinuation, including non-compliance, pregnancy, and medical complications, were limited to patients with psoriatic arthritis (13%, n = 3). However, the rate was 0% for ankylosing spondylitis (p = 0.544), possibly due to factors such as a younger average age and higher female representation in psoriatic arthritis populations. Documentation gaps, as indicated by the unexplained cessation of records, were observed uniformly across all disease types (13.0% vs. 11.1%, p = 1.000).

**Table 13:** Reasons for discontinuation stratified by key variables.

Reason for discontinuation	Overall (n=32)	PsA (n=23)	AS (n=9)	p-value^a^
Lack of efficacy, n (%)	19 (59.4)	12 (52.2)	7 (77.8)	0.238
Adverse events, n (%)	6 (18.8)	5 (21.7)	1 (11.1)	0.652
Other reasons, n (%)	3 (9.4)	3 (13.0)	0 (0.0)	0.544
Not specified, n (%)	4 (12.5)	3 (13.0)	1 (11.1)	1.000

^a^Fisher’s exact test comparing PsA vs AS.

**Figure 1:**
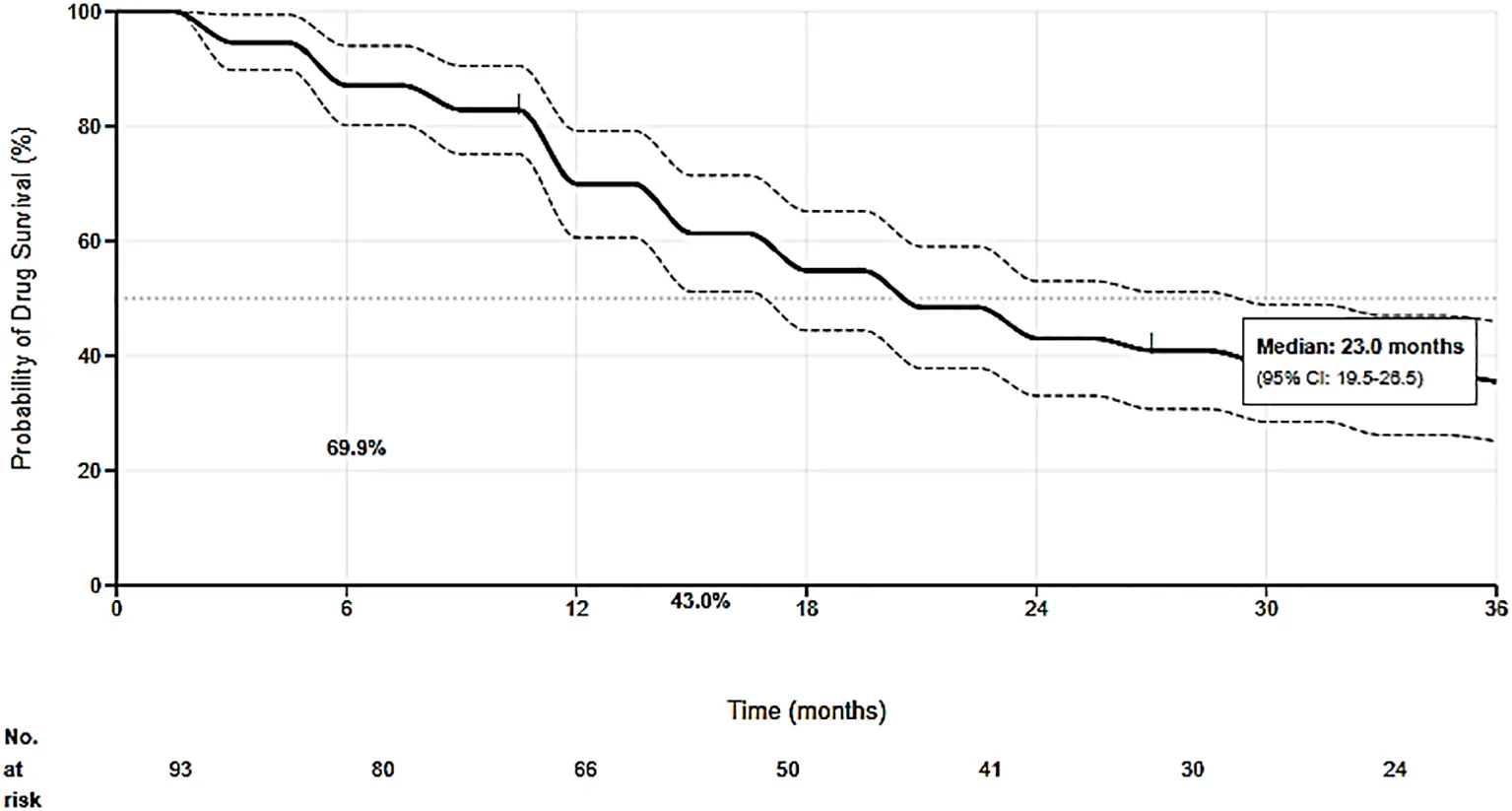
Kaplan-Meier survival curves for the ixekizumab drug survival. Kaplan-Meier curve showing the probability of ixekizumab survival in 93 patients with spondyloarthropathies. The solid line represents the survival probability, with 95% confidence intervals shown as dashed lines and shaded areas. Vertical marks indicate the censored observations. The numbers at risk are shown below the x-axis. The median drug survival was 23.0 months (95% CI: 19.5-26.5), with 69.9% (95% CI: 60.6-79.2%) of patients continuing treatment at 12 months and 43.0% (95% CI: 33.0-53.0%) at 24 months.

**Figure 1** Shows the overall ixekizumab drug survival of our cohort. There was a biphasic discontinuation pattern on the Kaplan-Meier curve, characterized by a sharp initial steep drop, where 30.1% of participants discontinued by 12 months, followed by a more gradual attrition. The median drug survival was 23.0 months (95% CI: 19.5-26.5), with survival rates of 69.9% and 43.0% at 12 and 24 months, respectively. It is striking that the steepest decline occurred between 9 and 12 months, suggesting a critical period for treatment adherence. Following year one, the survival curve displayed relative stability, suggesting that most patients who managed to endure the demanding early phases were likely to continue treatment over time. This indicates that enduring this challenging phase facilitates long-term retention while emphasizing the importance of close monitoring in the first year to enhance outcomes during therapy.

## Discussion

The drug survival rates in our study (69.9% at 12 months and 43.0% at 24 months) are notable for their alignment with those of significant international registries. The BIOBADASER 3.0 registry reported a nearly identical 12-month survival rate of 69.5% (95% CI: 64.0-74.3) among 335 patients, with retention rates of 71.3% in patients with psoriatic arthritis and 63.8% in those with axial spondyloarthritis (10, 24, 25). Italian multicenter data corroborate these findings, revealing a 38-month cumulative survival rate of 43.8%, which is almost exactly the same as the results after two years (26). The Corrona Psoriasis Registry further substantiates these trends by documenting a biologic-experienced patient cohort with a retention rate of 65% at the end of year one, where those treated with ixekizumab had a markedly lower discontinuation risk (HHR = 0.36) relative to that of TNF inhibitors (27–29). However, the real-world retention rates starkly differ from clinical trial figures, where continuation beyond three years within the COAST program exceeded 86% (30, 31). This efficacy-effectiveness gap reflects the inherent differences between controlled trial populations and real-world patients, who often have a greater comorbidity burden, less treatment experience, and less stringent monitoring protocols. Meta-analyses confirm this pattern across biologics, with pooled 12-month survival estimates ranging from 70-85% for first-line therapy, dropping to 60-75% for second-line use (27, 32, 33).

Comparative effectiveness analyses revealed significant differences within the IL-17 inhibitor class. Network meta-analyses comprising six Phase III studies with a total of 1, 733 subjects demonstrated robust efficacy (34, 35). with ASAS40 response rates achieving a relative risk of 2.12 (95% CI 1.75–2.56, p<0.01) compared to placebo (34, 36). Real-world data from registries are increasingly reporting greater outcomes for ixekizumab than for secukinumab, as noted in the Danish DERMBIO registry, wherein bionaive discontinuation rates were 0% for ixekizumab and 23.5% for secukinumab at the year mark (33, 37). The Czech BIOREP registry reported that brodalumab had the highest probability of drug survival, followed by ixekizumab and secukinumab. However, data on brodalumab are limited because of psychiatric safety concerns stemming from preliminary studies (25).Korean registries showed that the 3-year retention rates for secukinumab and ixekizumab were comparable at 62% each, with no marked difference (HR = 0.828, 95% CI 0.303-2.257, p = 0.712) (38). Within-class switching studies offer further clarity; among those who switched from secukinumab to ixekizumab, 62.5% demonstrated improvement in DAPSA, and half showed clinically meaningful axial improvement (26, 39, 40).

Recent head-to-head comparisons between psoriatic arthritis and axial spondyloarthritis have shown no discernible difference in the survival of IL-17 inhibitor drugs, contradicting early predictions. Almost identical survival curves were observed in a landmark Leeds study involving 228 patients; multivariate adjustment did not alter the hazard ratio of 0.92 (95% CI, 0.63-1.33) (29, 41). This implies that IL-17 inhibition may provide more consistent efficacy across spondyloarthritis subtypes, in contrast to previous TNF inhibitor data, which demonstrated superior retention in patients with PsA (41). Mechanistic insights explain these patterns. Verywell Health axial disease exhibits a strong response through entheseal IL-17 pathways, whereas psoriatic arthritis benefits from dual skin and joint targeting, with 85% of patients having concurrent psoriasis (42–45). Although HLA-B27 positivity rates vary significantly (85–95% in AS versus 20% in PsA), both conditions exhibit similar activation of the IL-17 pathway, indicating the potential for similar therapeutic responses (43, 46).

Furthermore, our findings, which identify third-line therapy as a predictor of discontinuation, align with the compelling evidence of diminishing returns across sequential biologics. The Australian OPAL registry data illustrate the phenomenon starkly: the median persistence drops from 96 months (first line) to 19 months (second line) and then to 15 months (third line). An analysis of Swedish registries conducted on 2, 590 patients quantified these differences, showing hazard ratios of 1.47 (95% CI 1.13-1.91) for second-line and 1.65 (95% CI 1.18-2.30) for third-line discontinuation relative to first-line therapy (47, 48).

This progressive decline captures both patient selection and the underlying biological factors at play. Baseline characteristics for third-line patients typically include a longer duration of disease, greater activity levels at baseline, and more treatment failures from prior lines of therapy (10, 47). Finnish registry data showed that BASDAI response rates plummeted from 52% with the first TNF inhibitor to only 25% with the second, demonstrating the difficulties associated with sequential therapy (47, 49). Notably, 76% of secukinumab use occurs as third- or later-line therapy, potentially biasing drug-specific comparisons (47, 50).

The treatment-emergent adverse event rate of 7.5% in our study aligns precisely with integrated safety analyses across 8, 228 patients, representing 20, 895.9 patient-years of ixekizumab exposure (18, 20, 51). The discontinuation rate due to lack of efficacy for psoriasis (7.5%), psoriatic arthritis (8.2%), and axial spondyloarthritis (7.1%) remained constant, with an incidence rate (IR) of 2.9 per 100 patient-years. Our cohort’s lack of significant adverse events reflects the broader safety profile. However, previous investigations revealed that serious infection rates remain low (IR ≤1.3/100 PY) and major adverse cardiovascular events are rare (IR ≤0.6/100 PY) (18, 52).

Longitudinal data concerning TEAEs over five years show a temporal improvement, notably a reduction in overall TEAEs from 88.9/100 PY in year one to 63.2/100 PY in year five (18, 52). Injection site reactions, the most frequent TEAE, also exhibited this trend, decreasing significantly from 16.5/100 PY to 1.7/100 PY during this timeframe. Through post-marketing surveillance within the PSOLAR, FAERS, and EudraVigilance databases, no new safety signals have been identified, with Candida infections accounting for the primary class-specific risk, occurring at a rate of 1.9-2.0/100 PY (51, 53).

### Study strengths and limitations

Our study adds to the body of real-world evidence regarding the use of ixekizumab for treatment in the effectiveness and safety domains for a Saudi population, fills a significant void considering that 82% of participants in existing clinical trials were White, and employs rigorous statistical methods, including Kaplan-Meier survival analysis and Cox regression modeling, on longitudinal data obtained from electronic health records within two tertiary care centers. However, the study’s retrospective nature comes with issues such as selection bias due to dependency on medical record completeness, HLA-B27 was not documented in 88.2% of cases and baseline CRP was available in only 60% of patients, which may have underpowered the univariable CRP analysis in **Table 10**, limited enrollment affecting power resulting in small sample sizes for subgroup analyses (such as the fourth-line therapy group containing only two patients), and gaps in documentation such as lack of detailed reasoning for discontinuation in 12.5% of cases where no rationale was provided alongside absence of clinical justification. Furthermore, inconsistent recording of validated disease activity scores at 12 and 24 months, together with heterogeneous prior biologic histories, small subgroup sizes and incomplete documentation of disease duration before ixekizumab initiation, limited our ability to perform robust survival analyses stratified by remission/low disease activity, prior biologic class or pre-treatment disease duration. Regardless of these limitations, the overarching outcomes remained consistent with those captured by international registries, which bolsters their external validity and generalizability, illustrating the substantial real-world evidence from populations underrepresented in spondyloarthropathy research.

## Conclusion

Our study demonstrates the use of ixekizumab in spondyloarthropathies, as well as its safety and effectiveness in typical clinical practice. It captures real-world scenarios rather than controlled settings, where more advanced cases are treated, including evolving multi-morbidities and failed prior biologic schemes. In contrast to the findings from tightly monitored clinical trials, we observed lower retention rates of 69.9% at 12 months and 43% at 24 months. However, our results corroborate these retention values, alongside a median drug survival of 23 months.

The observation that patients with psoriatic arthritis had greater drug survival than those with ankylosing spondylitis is indicative of disease subtype differences. The observed differential retention between psoriatic arthritis and ankylosing spondylitis may reflect underlying differences in disease phenotype and IL-17A-driven pathology, as psoriatic arthritis often involves both peripheral joint and skin disease, whereas ankylosing spondylitis predominantly affects the axial skeleton.

These findings highlight the need for timely intervention following underlying event management aimed at preventing adverse events, which proactively improves outcomes after optimizing ixekizumab therapy, focusing on early strategy deployment.

The favorable safety profile of ixekizumab in our cohort, with only 7.5% of patients experiencing treatment-emergent adverse events and no serious adverse events, supports its use in diverse global populations. However, the strong association of any adverse event with discontinuation of therapy underscores the importance of patient education, expectation management, and careful observation during the early stages of treatment.

These findings justify modifying standard methods for managing spondyloarthropathy by placing more emphasis on disease subtype, previous therapies tried, and other characteristics specific to the patient’s profile. To further refine clinical outcomes across various settings with increased sample sizes and extended follow-up durations, additional prospective studies are needed to confirm these insights and adjust treatment frameworks.

## Data Availability

The de-identified data that support the findings of this study are available from the corresponding author upon reasonable request. The data are not publicly available due to privacy and institutional restrictions.
